# Randomized Trial of the Choosing Wisely Consumer Questions and a Shared Decision-Making Video Intervention on Decision-Making Outcomes

**DOI:** 10.1177/0272989X231184461

**Published:** 2023-07-05

**Authors:** Danielle Marie Muscat, Rachel Thompson, Erin Cvejic, Jenna Smith, Edward Hoi-fan Chang, Marguerite Tracy, Joshua Zadro, Robyn Lindner, Kirsten J. McCaffery

**Affiliations:** Faculty of Medicine and Health, School of Public Health, Sydney Health Literacy Lab, University of Sydney, New South Wales, Australia; Faculty of Medicine and Health, School of Public Health, Wiser Healthcare, University of Sydney, New South Wales, Australia; Faculty of Medicine and Health, School of Public Health, Sydney Health Literacy Lab, University of Sydney, New South Wales, Australia; Faculty of Medicine and Health, School of Public Health, Wiser Healthcare, University of Sydney, New South Wales, Australia; Faculty of Medicine and Health, School of Health Sciences, University of Sydney, New South Wales, Australia; Faculty of Medicine and Health, School of Public Health, Sydney Health Literacy Lab, University of Sydney, New South Wales, Australia; Faculty of Medicine and Health, School of Public Health, University of Sydney, New South Wales, Australia; Faculty of Medicine and Health, School of Public Health, Sydney Health Literacy Lab, University of Sydney, New South Wales, Australia; Faculty of Medicine and Health, School of Public Health, Wiser Healthcare, University of Sydney, New South Wales, Australia; Faculty of Medicine and Health, School of Public Health, Sydney Health Literacy Lab, University of Sydney, New South Wales, Australia; Faculty of Medicine and Health, School of Public Health, Sydney Health Literacy Lab, University of Sydney, New South Wales, Australia; Faculty of Medicine and Health, School of Public Health, Institute for Musculoskeletal Health, University of Sydney, New South Wales, Australia; NPS Medicinewise, New South Wales, Australia; Faculty of Medicine and Health, School of Public Health, Sydney Health Literacy Lab, University of Sydney, New South Wales, Australia; Faculty of Medicine and Health, School of Public Health, Wiser Healthcare, University of Sydney, New South Wales, Australia

**Keywords:** shared decision making, health literacy, question prompt list, medical overuse, low-value care, patient participation, decision making

## Abstract

**Background:**

Despite widespread use, there are few studies evaluating the consumer Choosing Wisely questions.

**Methods:**

We evaluated the impact of the Choosing Wisely questions on consumers’ decision-making outcomes. Adults living in Australia were presented with a hypothetical low-value care scenario. Using a 2×2×2 between-subjects factorial design, they were randomized to either the Choosing Wisely questions (“Questions”), a shared decision-making (SDM) preparation video (“Video”), both interventions, or control (no intervention). Primary outcomes were 1) self-efficacy to ask questions and be involved in decision-making and 2) intention to engage in SDM.

**Results:**

A total of 1,439 participants (45.6% with “inadequate” health literacy) were eligible and included in the analysis. Intention to engage in SDM was higher in people randomized to the Video (mean difference [MD] = 0.24 [scale 0–6], 95% confidence interval [CI]: 0.14, 0.35), Questions (MD = 0.12, 95% CI: 0.01, 0.22), and both interventions (MD = 0.33, 95% CI: 0.23–0.44, *P* < 0.001, *d* = 0.28) compared with control. Combining interventions had a greater impact than presenting the Questions alone (MD = 0.22, 95% CI: 0.11, 0.32; *P* < 0.001). Those who received the Video or both interventions reported lower intention to follow the low-value treatment plan without further questioning (all *P* < 0.05) and more positive attitudes toward SDM (all *P* < 0.05) compared with control. Intervention acceptability was high in all study arms (>80%), but proactive access was low (1.7%–20.8%). Compared with control, participants who received one or both interventions asked more questions that mapped to the Choosing Wisely questions (all *P* < .001). There were no main effects of either intervention on self-efficacy or knowledge.

**Conclusions:**

The Choosing Wisely questions and a video to promote SDM may improve intention to engage in SDM and support patients in identifying questions that align with the Choosing Wisely campaign (with some additional benefits of the video intervention).

**Trial registration:**

ANZCTR376477

**Highlights:**

- We conducted a randomized controlled trial online with adults living in Australia to test the effectiveness of the consumer Choosing Wisely questions and a shared decision-making (SDM) preparation video.
- Both interventions improved intention to engage in SDM and supported participants to identify questions that align with the Choosing Wisely campaign.
- There were some additional benefits of the Video intervention in reducing willingness to accept low-value treatment for low-back pain without asking questions; however, neither intervention changed participants’ self-efficacy to ask questions and be involved in decision-making nor affected perceptions of preparedness to engage in SDM or knowledge of rights to be involved in health care decision-making.
- The simple, low-cost nature of the interventions may make them appropriate for implementation within a suite of approaches to address low-value care at a population level.

There is an increasing recognition of the need to reduce unnecessary and low-value medical care and decrease waste in the health sector. Unnecessary and potentially harmful health service use accounts for a significant proportion of total health expenditure.^1^ One initiative that has gained momentum worldwide is Choosing Wisely®, a campaign that has now been adapted and implemented in more than 20 countries.^2^ The campaign seeks to encourage clinicians and patients to talk about medical tests and procedures that may be unnecessary and, in some instances, can cause physical and psychological harm.^2^ While acknowledging that it is often challenging to have conversations about unnecessary tests and treatments, leaders of the campaign consider communication between clinicians and patients during routine clinical encounters a key mechanism for change.^2^ To this end, 1 of the 4 key pillars of the Choosing Wisely International Roundtable’s Framework for Patient and Public Engagement in Choosing Wisely Campaigns was to support shared decision-making (SDM) at the clinical level.^3^

As part of the original Choosing Wisely campaign, 5 questions were developed for patients to ask healthcare providers to support better conversations about unnecessary tests, medications, and procedures.^4^ The questions are publicly available and have been promoted for use nationally and internationally in several forms and languages.

In Australia, the Choosing Wisely Australia® 5 Questions (Box 1) have been promoted for their “potential to facilitate better conversations between healthcare providers and consumers.”^5^ However, annual evaluation surveys conducted by Choosing Wisely Australia® have primarily assessed consumer awareness of the questions and the number of downloads of a resource explaining the questions, rather than their impact on people’s self-efficacy, ability, and intention to use the questions to engage in SDM.^5^ This focus on reach rather than impact is mirrored internationally,^6^ with increasing calls for more thorough evaluations to generate cross-national insights on tools to advance the Choosing Wisely approach.^7^ Despite this, a systematic review of interventions designed to reduce medical care identified as low-value by Choosing Wisely published in 2021 continued to identify few randomized trials internationally and a “dearth of studies” evaluating the impact of consumer-based Choosing Wisely interventions.^8^

**Box 1:** The Choosing Wisely Australia® 5 Questions

1. Do I really need this test, treatment or procedure? 2. What are the risks? 3. Are there simpler, safer options? 4. What happens if I don’t do anything? 5. What are the costs?

©NPS Medicinewise Ltd. Reproduced with permission. Visit www.choosingwisely.org.au

Since then, a single prospective, randomized controlled trial in 3 intensive care units in Western Australia evaluated the impact of providing families with the Choosing Wisely questions as printed prompts prior to a family meeting. The primary outcome was family perceived involvement in decision-making. Of the 60 families who participated in the study, most (87.1% control, 79.3% intervention; *P* = 0.334) reported feeling “very included” in decision-making,^9^ with no difference in primary or secondary outcomes and minimal uptake of the questions by the intervention group.^9^ Another qualitative study with 22 Australian general practice patients suggested that participants considered the Choosing Wisely questions to be valuable in guiding patients to reflect on decisions with their doctor and to take greater responsibility for their health care decisions.^10^ However, participants felt that use of the questions in their own health care was not necessary for several reasons: they perceived doctors made the “right” decision for their management (in this case, requesting a computed tomography scan); they had an established relationship with, and trust in, their general practitioner (GP); the GP had communicated about benefits and harms already; and/or because the “doctor knew best.” Several participants also noted that an explanation of the 5 Choosing Wisely questions would be needed for the questions to be used.^10^ Other findings from Choosing Wisely Australia® suggested that people may continue to feel that they do not have permission to ask questions.^5^ Together, this existing evidence suggests that the 5 questions alone may not be sufficient for enabling patient question asking. However, their impact on people’s self-efficacy, ability, and intention to engage in SDM is yet to be formally evaluated.

The aim of this study was to evaluate the impact of the Choosing Wisely Australia® 5 Questions resource (“Questions”). The primary outcomes were 1) self-efficacy to ask questions and be involved in decision-making and 2) intention to engage in SDM. Secondary outcomes included intention to follow the treatment plan without further questioning, knowledge of the patients’ rights regarding SDM, positive attitude toward SDM, preparedness for SDM, acceptability of interventions, and proactive intervention use.

## Methods

The study methods are described in our published protocol.^11^ Ethical approval was obtained from the University of Sydney Human Research Ethics Committee (protocol No. 2018/965), and the trial was registered with the Australia New Zealand Clinical Trials Registry (trial No. 376477).

### Study Design

This study was conducted online between November and December 2019 using the Qualtrics survey platform. We used a 2×2×2 between-subjects factorial design (Video: yes, no × Questions: yes, no × health literacy: adequate, inadequate). We aimed to recruit equal numbers of participants with inadequate and adequate health literacy by oversampling participants who had less than a university degree level of education compared with those with a university degree level of education or greater (using a 70:30 ratio, respectively). However, this article reports on the intervention effects only; preplanned analyses related to the role of health literacy will be reported separately.

### Participants, Recruitment, and Consent

Participants were Australian citizens or permanent residents aged 18 y or older who self-reported sufficient English language skills to complete questionnaires in English. Participants were identified, prescreened for eligibility, and invited to consider participation by Dynata, a social research company with a database of >600,000 people.

Interested participants were directed to an online survey where they provided written informed consent to participate and were presented with a hypothetical low-value healthcare scenario that asked them to imagine being in a situation in which they have non-specific low-back pain and stable pain/symptoms (see Box 2).

**Box 2:** Hypothetical Back Pain Scenario

You have had lower back pain for about one month; it has not improved or become worse. You did not have an accident to cause the pain; it just began and has not gone away. You go to your doctor to get advice on what is causing it and what can help with the pain. The doctor recommends that you have a scan to help figure out what is causing the pain, and gives you a medicine prescription.

### Randomization and Intervention Description

Randomization was undertaken via an automated function in the survey platform using an equal allocation ratio and stratification by participant health literacy (adequate, inadequate), yielding 4 trial arms in each health literacy subgroup: 1) “Questions,” 2) “Video,” 3) both interventions, and 4) control (no intervention). Participants were not blinded to their assigned intervention.

#### Preparation Video

We developed a short 3-min video intended to prepare patients for question asking and SDM. The video script (available in Muscat et al 2019.^11^) integrated recommendations for SDM preparation as outlined by Joseph-Williams and colleagues.^12^ This included making explicit what SDM is, what to expect, and why it is appropriate; explaining that there are 2 experts in the clinical encounter; challenging attitudes that there are right and wrong decisions; reassuring patients that participation in decision-making will not result in retribution and confirming that clinicians want patient participation; and building patients’ belief in their ability to take part. The transcript was developed at a grade 5 readability level and incorporated techniques to reduce cognitive burden.^11^

#### Choosing Wisely questions

The Choosing Wisely Australia® 5 Questions (see Box 1 above) were presented to participants via a 1-page document that was developed and co-branded by Choosing Wisely Australia® and NPS Medicinewise. This resource lists and elaborates on the questions and provides additional guidance on their rationale and use. This resource has a grade 9 readability level and is publicly available from the Choosing Wisely Australia® website. See Appendix A.

#### Implementation of interventions

The interventions were displayed to participants within the survey platform. To encourage attention to the intervention, a timer was added to the pages displaying the Video (3 min) and 5 Questions resource (1 min), preventing participants from progressing to the next survey page until the specified time had elapsed. For those receiving both interventions, the Video was presented before the Choosing Wisely Questions. Participants were not prevented from exposure to any other care or interventions prior to or during the study.

### Data Collection

Study data were collected via surveys administered immediately before (pre), immediately after (post), and 2 wks fter (follow-up) exposure to the relevant intervention(s). The primary time point was immediately post-intervention.

### Outcomes and Measures

Primary and secondary outcome measures, including how and when they were assessed, are reported in Table 1 and our published protocol.^11^ Participants were also asked to report basic demographic and health information, including age, gender, state of residence, language spoken at home, education status, employment status, private health insurance status, confidence in filling out medical forms,^13^ who is usually involved in decision-making related to their health, and experience and perceived knowledge of low-back pain. Health literacy was assessed by the Newest Vital Sign (NVS^14^), with participants categorised as inadequate (score 0–3 on NVS) or adequate health literacy (score 4–6 on NVS). The NVS is an objective, performance-based measure of health literacy skills, which has been used on other online studies.^15^

**Table 1:** Outcomes and Measurement

	Outcome	Measure	Pre	Post	Follow-up
Primary	Self-efficacy to ask questions	Single item adapted from Bandura’s self-efficacy theory.^16^ Participants were asked to rate their degree of confidence to ask questions of their health care provider by recording a number from 0 (*Cannot do at all*) to 100 (*Highly certain can do*).	x	x	x
	Self-efficacy to be involved in health care decision-making	Single item adapted from Bandura’s self-efficacy theory.^16^ Participants were asked to rate their degree of confidence to be involved in decisions with their health care provider by recording a number from 0 (*Cannot do at all*) to 100 (*Highly certain can do*).	x	x	x
	Self-efficacy to ask questions and be involved in health care decision-making	Composite measure based on 2 individual items (see above)	x	x	x
	Intention to engage in shared decision-making	Validated, 3-item scale (Cronbach alpha = 0.8^17^) measuring participants’ 1) likelihood of engaging in shared decision-making, from *very unlikely* (−3) to *very likely* (+3); 2) odds of engaging in shared decision-making, from *very weak* (−3) to *very strong* (+3); and 3) agreement with the statement, “I intend to engage in shared decision-making,” from *total disagreement* (−3) to *total agreement* (+3). Total scores will be rescaled on a scale of 0–6 and the sum of the items divided by 3 to derive the total score of intention.	x	x	x
Secondary	Intention to follow the treatment plan recommended by the doctor without further questioning	A single item on a 10-point scale, adapted from previous research,^18^ assessing hypothetical intention to follow the treatment plan recommended by the doctor without further questioning: “Which best describes your intention to follow the treatment plan recommended by the doctor without asking further questions?” (1 = *Definitely will not* to 10 = *Definitely will*).	x	x	x
	Knowledge of patients’ health care rights	Four questions adapted from Halawany et al.^19^ and applied to the Australian Charter of Healthcare Rights (second edition).^20^ Participants were asked to indicate “Yes,”“No,” or “Unsure” to show whether they think the following are patient rights: 1) ask questions and be involved in open and honest communication, 2) make choices with your health care provider, 3) include the people who you want in planning and decision-making; 4) get clear information about your condition, including the possible benefits and risks of different tests and treatments. A foil question will be included to detect if participants are arbitrarily selecting “yes” to all questions. Scores are dichotomised into 1) all questions correct or 2) not all questions correct.	x	x	—
	Attitude toward shared decision-making	Three-item scale adapted from Dormandy et al.^21^ assessing participants’ perceptions of shared decision-making as beneficial/not beneficial, worthwhile/not worthwhile, and important/unimportant. Each item has 7 response options, forming a scale from 3 to 21. Scores were recoded such that higher scores indicate more positive attitudes toward shared decision-making. Participants responding with the highest possible score on all 3 questions were classified as having positive attitudes.	—	x	—
	Preparedness for shared decision-making	Modified, 8-item version of the Preparation for Decision Making Scale (PrepDM).^22^ The PrepDM scale was developed to assess a participants’ perception of how useful a decision support intervention is in preparing them to communicate with their practitioner at a consultation visit and to make a health decision. Items were scored on a Likert-type scale from 1 to 5, from *Not at all* (1) to *A great deal* (5), with higher scores indicating higher perceived level of preparation for decision-making. Items were summed and the total score divided by 8.	—	x	—
	Acceptability (arms 1–3 only)	Adapted from Shepherd et al.,^23^ participants were asked to rate if they would 1) recommend the [intervention] to others and 2) use the [intervention] again on a 4-point scale ranging from 1 (*Definitely not*) to 4 (*Yes, definitely*) (Shepherd et al.).^23^ Recommendations are dichotomized into *would recommend* (3 and 4) and *would not recommend* (1 and 2).	—	x	—
	Indicator of proactive intervention use (arms 1–3 only)	We assessed the proportion of participants who clicked on a link to their intervention.	—	x	x
	Health care questions	Participants were asked to write down 5 questions that they would ask the doctor given the hypothetical health care scenario. The content of individual responses were analyzed via content analysis using inductive and deductive approaches (see below). The mean number of questions that map onto the Choosing Wisely 5 Questions was calculated.	—	x	x

## Analysis

### Quantitative Data Analysis

Quantitative data analyses were conducted using Stata/IC v16.1 (StataCorp, College Station, TX) by a study statistician blinded to the intervention allocation of participants and their level of health literacy. Primary and secondary outcome data were analyzed as intention to treat using linear regression for continuous outcomes and logistic regression for dichotomous categorical outcomes. Dichotomous variables representing the study factors (Video: yes, no × Questions: yes, no × health literacy: adequate, inadequate) and their interactions were included in models as between-subjects fixed effects, controlling for preintervention values (where available). Pairwise comparisons of estimated marginal means between the intervention arms and control were also explored, with Cohen’s *d* provided as a measure of effect size. Outcome data collected during the immediate post and follow-up survey were analyzed in separate models. A *P* value of 0.05 was set as the threshold for statistical significance. In line with our published protocol,^11^ multiple imputation was used to impute missing responses on primary outcomes for participants who completed the initial (pre- and post-) surveys but did not return to complete the 2-wk follow-up survey. The conclusions drawn from these analyses were comparable to complete case analysis, so in the interest of brevity, only complete case analysis is reported in text. Results from multiple imputation analyses are provided in Appendix B.

### Sample Size

A sample size calculation was conducted based on the primary outcome of intention.^11^ Previously published values and pilot data informed the anticipated effect.^17^ We calculated that 162 subjects per intervention group would be sufficient to detect small effect sizes (*d* = 0.10 for the Questions; *d* = 0.20 for the Video, and their interaction) with 80% power at a *P* value of 0.05 in primary analyses. Therefore, we aimed to recruit a total sample of 1,432 participants (including 50% with inadequate health literacy; *n* = 179 participants per intervention group), to allow for approximately 10% dropout between pre- and postintervention measures.

### Content Analysis

We used summative content analysis to code the open-text responses participants provided when asked what they would ask the doctor given the hypothetical healthcare scenario.^24^ We coded the data to assess the frequency of questions matching or close to the Choosing Wisely Australia 5 Questions. For each of the questions, participants were given a code of “1” (i.e., Choosing Wisely Question was among participant responses) or “0” if not. The total number of responses that mapped to the Choosing Wisely 5 questions per participant immediately, and 2 weeks post-intervention, was quantitatively compared using negative binomial regression including the study factors and their interactions (as described above). See Appendix C for additional information.

## Results

**Figure 1:**
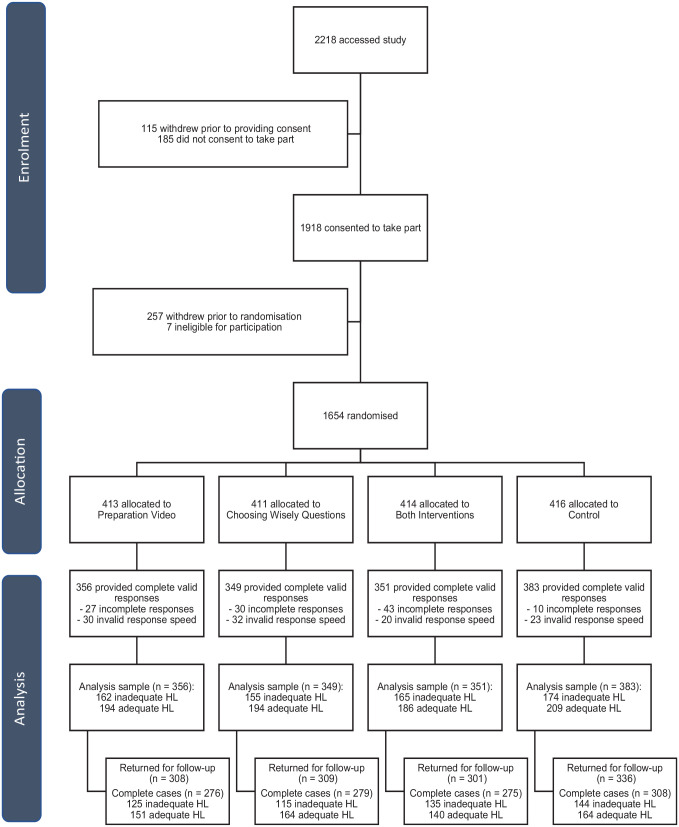
Participant flow diagram.

Of the 1,918 people who consented to take part in the study, 1,654 were randomised to 1 of the 4 study arms. Of those, 1,439 participants (87%) provided complete responses that were deemed valid due to completion time (not too fast [>210 s control arm, >330 s Questions arm, >480 s Video arm, >570 s Questions and Video arm] or too slow [<2 h]) and were included in the final analysis. These time cutoffs were chosen post hoc by determining an absolute minimum or maximum time for engagement with the content of the survey, due to concerns about the quality of fast or slow responses (Figure 1).

At 2-wk follow-up, 1,254 participants (87.1% of analysis sample; 75.8% of initially randomized sample) completed the questionnaire. Of these, 16 began but did not complete the survey, 44 had invalid response speeds, and 54 had partially incomplete data (i.e., did not respond to all follow-up questions).

### Sample Characteristics

Descriptive characteristics of the analysis sample are shown in Table 2. The greatest proportion of participants (27.5%) were aged between 31 and 45 y, and 48.8% identified as female. Most reported speaking English at home (93%), and one-third of the sample was employed full-time (33.4%). Inadequate health literacy was observed in 45.6% of the sample.

**Table 2:** Descriptive Characteristics of the Analysis Sample (*N* = 1,439) by Randomized Intervention Group^a^

	Video (*n* = 356)	Questions (*n* = 349)	Both Interventions (*n* = 351)	Control (*n* = 383)	Total
Age (y)					
18–30	76 (21.3)	70 (20.1)	74 (21.1)	88 (23.0)	308 (21.4)
31–45	96 (27.0)	87 (24.9)	106 (30.2)	107 (27.9)	396 (27.5)
46–60	80 (22.5)	94 (26.9)	74 (21.1)	71 (18.5)	319 (22.2)
61–75	85 (23.9)	91 (26.1)	80 (22.8)	99 (25.9)	355 (24.7)
Gender					
Male	174 (48.9)	180 (51.6)	178 (50.7)	202 (52.7)	734 (51.0)
Female	181 (50.8)	168 (48.1)	173 (49.3)	180 (47.0)	702 (48.8)
Nonbinary	1 (0.3)	1 (0.3)	0 (0.0)	0 (0.0)	2 (0.1)
Prefer not to say	0 (0.0)	0 (0.0)	0 (0.0)	1 (0.3)	1 (0.1)
English as main language spoken at home	330 (92.7)	320 (91.7)	324 (92.3)	364 (95.0)	1,338 (93)
Education					
University degree	107 (30.1)	96 (27.5)	105 (29.9)	98 (25.6)	406 (28.2)
Diploma or certificate	108 (30.3)	98 (28.1)	106 (30.2)	120 (31.3)	432 (30.0)
High school certificate or trade apprenticeship	101 (28.4)	111 (31.8)	108 (30.8)	114 (29.8)	434 (30.2)
School certificate or less	40 (11.2)	44 (12.6)	32 (9.1)	51 (13.3)	167 (11.6)
Employment status					
Working full-time	105 (29.5)	122 (35.0)	121 (34.5)	133 (34.7)	481 (33.4)
Working part-time	71 (19.9)	64 (18.3)	59 (16.8)	63 (16.4)	257 (17.9)
Not currently employed	32 (9.0)	27 (7.7)	27 (7.7)	42 (11.0)	128 (8.9)
Family caring/home duties	35 (9.8)	37 (10.6)	36 (10.3)	39 (10.2)	147 (10.2)
Retired	95 (26.7)	77 (22.1)	95 (27.1)	91 (23.8)	358 (24.9)
Studying full-time	15 (4.2)	20 (5.7)	13 (3.7)	9 (2.3)	57 (4.0)
Prefer not to answer	3 (0.8)	2 (0.6)	0 (0.0)	6 (1.6)	11 (0.8)
Private health insurance	190 (53.4)	193 (55.3)	189 (53.8)	223 (58.2)	795 (55.2)
Confidence filling out medical forms					
Extremely	168 (47.2)	183 (52.4)	181 (51.6)	187 (48.8)	719 (50.0)
Quite a bit	127 (35.7)	122 (35.0)	114 (32.5)	128 (33.4)	491 (34.1)
Somewhat	50 (14.0)	36 (10.3)	45 (12.8)	55 (14.4)	186 (12.9)
A little bit	4 (1.1)	6 (1.7)	9 (2.6)	11 (2.9)	30 (2.1)
Not at all	7 (2.0)	2 (0.6)	2 (0.6)	2 (0.5)	13 (0.9)
Involvement in health care decision-making related to their health					
Self	315 (88.5)	314 (90.0)	324 (92.3)	341 (89.0)	1,294 (89.9)
Doctor	277 (77.8)	300 (86.0)	290 (82.6)	318 (83.0)	1,185 (82.3)
Partner or spouse	124 (34.8)	130 (37.2)	130 (37.0)	130 (33.9)	514 (35.7)
Adult child	18 (5.1)	2 (0.6)	10 (2.8)	5 (1.3)	35 (2.4)
Friend	7 (2.0)	4 (1.1)	6 (1.7)	8 (2.1)	25 (1.7)
Brother(s) or sister(s)	7 (2.0)	2 (0.6)	9 (2.6)	5 (1.3)	23 (1.6)
Another relative	5 (1.4)	2 (0.6)	3 (0.9)	8 (2.1)	18 (1.3)
Professional carer	4 (1.1)	1 (0.3)	1 (0.3)	4 (1.0)	10 (0.7)
Court-appointed guardian	1 (0.3)	1 (0.3)	0 (0.0)	1 (0.3)	3 (0.2)
Parent(s)	37 (10.4)	35 (10.0)	37 (10.5)	36 (9.4)	145 (10.1)
Back pain history	121 (34.0)	119 (34.1)	135 (38.5)	137 (35.8)	512 (35.6)
Back pain knowledge					
Not much at all	76 (21.3)	67 (19.2)	72 (20.5)	80 (20.9)	295 (20.5)
A little	216 (60.7)	214 (61.3)	226 (64.4)	244 (63.7)	900 (62.5)
A lot	64 (18.0)	68 (19.5)	53 (15.1)	59 (15.4)	244 (17.0)
Health literacy (newest vital sign)					
Inadequate	162 (45.5)	155 (44.4)	165 (47.0)	174 (45.4)	656 (45.6)
Adequate	194 (54.5)	194 (55.6)	186 (53.0)	209 (54.6)	783 (54.4)
Self-efficacy to ask questions and be involved in health care decision-making, $\bar{x}$ (*s*)	83.0 (16.8)	84.1 (15.8)	82.7 (17.0)	81.5 (17.3)	82.8 (16.8)
Intention to engage in shared decision-making, $\bar{x}$ (*s*)	4.8 (1.1)	4.8 (1.2)	4.7 (1.2)	4.7 (1.2)	4.7 (1.2)
Intention to follow the treatment plan without further questioning, $\bar{x}$ (*s*)	7.4 (2.3)	7.7 (2.1)	7.2 (2.3)	7.3 (2.3)	7.4 (2.3)
Accurate knowledge of patients’ rights in regard to shared decision-making	287 (80.6)	299 (85.7)	295 (84.0)	320 (83.6)	1,201 (83.5)

a Data are displayed as n (%) unless otherwise indicated.

### Primary Outcomes

Descriptive statistics for outcome measures, stratified by study arm, are presented in Table 3.

**Table 3:** Descriptive Statistics for Outcome Measures (Displayed as Estimated Marginal Means [Unless Otherwise Indicated] with 95% Confidence Intervals) and Pairwise Comparisons to Control (Displayed as Estimated Mean Difference [Unless Otherwise Indicated] with 95% Confidence Intervals and *P* Value), Immediately Postintervention and at Follow-up Stratified by Study Arm

Outcome Measure	Study Arm						Control
	Video		Questions		Both Interventions		
	Estimate	Versus Control	Estimate	Versus Control	Estimate	Versus Control	
Postintervention							
Self-efficacy to ask questions and be involved in health care decision-making (0 to 100)	84.7 (83.8, 85.6)	0.3 (−1.0, 1.6); *P* = 0.67	84.8 (83.9, 85.7)	0.4 (−0.9, 1.6); *P* = 0.58	84.9 (83.9, 85.8)	0.4 (−0.9, 1.7); *P* = 0.52	84.5 (83.7, 85.4)
Intention to engage in shared decision-making (0 to 6)	5.06 (4.99, 5.14)	0.24 (0.14, 0.35); *P* < 0.001*	4.93 (4.86, 5.01)	0.12 (0.01, 0.22); *P* = 0.031	5.15 (5.08, 5.23)	0.33 (0.23, 0.44); *P* < 0.001*	4.82 (4.75, 4.89)
Intention to follow treatment plan without further questioning (0 to 10)	7.00 (6.81, 7.20)	−0.32 (−0.59, −0.05); *P* = 0.020*	7.32 (7.13, 7.52)	0.00 (−0.27, 0.27); *P* = 0.99	7.04 (6.85, 7.24)	−0.28 (−0.55, −0.01); *P* = 0.040*	7.32 (7.14, 7.51)
Accurate knowledge of patients’ health care rights (proportion)	0.87 (0.84, 0.89)	0.02 (−0.02, 0.05); *P* = 0.39	0.85 (0.82, 0.88)	0.00 (−0.04, 0.04); *P* = 0.94	0.84 (0.82, 0.87)	0.00 (−0.04, 0.03); *P* = 0.83	0.85 (0.82, 0.88)
Positive attitude toward shared decision-making (proportion)	0.57 (0.52, 0.63)	0.10 (0.03, 0.17); *P* = 0.007*	0.54 (0.49, 0.59)	0.06 (−0.01, 0.13); *P* = 0.089	0.57 (0.51, 0.62)	0.09 (0.02, 0.16); *P* = 0.013	0.48 (0.42, 0.53)
Preparedness for shared decision-making (1–5)	3.88 (3.78, 3.97)	N/A	3.92 (3.82, 4.02)	N/A	3.99 (3.89, 4.09)	N/A	N/A
Follow-up (2 wk)							
Self-efficacy to ask questions and be involved in health care decision-making (0 to 100)	84.5 (83.0, 86.0)	1.9 (−0.1, 4.0); *P* = 0.068	83.2 (81.7, 84.7)	0.7 (−1.4, 2.7); *P* = 0.53	82.9 (81.4, 84.4)	0.4 (−1.7, 2.5); *P* = 0.71	82.5 (81.1, 84.0)
Intention to engage in shared decision-making (0 to 6)	4.99 (4.88, 5.10)	0.13 (−0.03, 0.28); *P* = 0.10	4.88 (4.77, 4.99)	0.01 (−0.14, 0.17); *P* = 0.87	4.94 (4.53, 5.05)	0.08 (−0.08, 0.23); *P* = 0.33	4.86 (4.76, 4.97)
Intention to follow treatment plan without further questioning (0 to 10)	7.11 (6.85, 7.37)	−0.26 (−0.62, 0.11); *P* = 0.17	7.20 (6.94, 7.46)	−0.17 (−0.53, 0.20); *P* = 0.37	6.92 (6.66, 7.19)	−0.44 (−0.81, −0.08); *P\* = 0.016*	7.37 (7.11, 7.62)

* statistical significance (*P*<.05).

#### Self-efficacy to ask questions and be involved in healthcare decision-making

Self-efficacy to ask questions and be involved in healthcare decision-making regarding treatment for low-back pain was relatively high overall. There was no evidence of a main effect of the Questions, *F*(1, 1,430) = 0.26, *P* = 0.61; Video, *F*(1, 1,430) = 0.04, *P* = 0.85; or their interaction, *F*(1, 1,430) = 0.17, *P* = 0.68, immediately post-intervention. Similarly, there was no evidence of a main effect of the Questions, *F*(1, 1,129) = 0.30, *P* = 0.59; Video, *F*(1, 1,129) = 1.25, *P* = 0.26; or their interaction, *F*(1, 1,129) = 2.28, *P* = 0.13, at 2-wk follow-up. Pairwise comparisons indicated that all 3 of the active intervention conditions were no better than control at either time point (all pairwise comparisons *P* > 0.05; see Table 3).

#### Intention to engage in SDM

**Figure 2:**
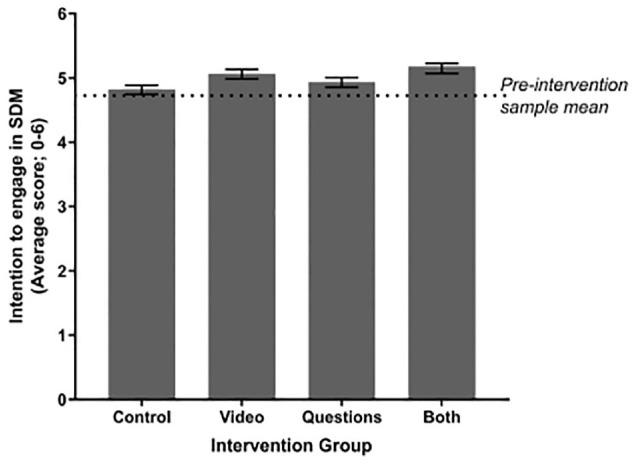
Estimated marginal means of intention to engage in shared decision-making (SDM) immediately postintervention, stratified by intervention group. The dotted horizonal line indicates the preintervention sample mean (4.72). All pairwise comparisons with control are statistically significant at *P* < 0.05. Error bars indicate 95% confidence intervals.

Immediately post-intervention, there was very strong evidence of a main effect of the Video on intention to engage in SDM regarding treatment for low-back pain, *F*(1, 1,430) = 36.70, *P* < 0.001, and strong evidence of a main effect of the Questions, *F*(1, 1,430) = 6.88, *P* = 0.009, on SDM intentions (see Figure 2). There was no intervention interaction effect, *F*(1, 1,430) = 0.06, *P* = 0.81.

Pairwise comparisons (Table 3) showed that all 3 of the active intervention conditions were significantly better than control (Video v. control: *P* < 0.001, *d* = 0.21; Questions v. control: *P* = 0.031, *d* = 0.10; both interventions v. control: *P* < 0.001, *d* = 0.28) and that combining the Video and Questions had a greater impact than presenting the Questions alone (mean difference = 0.22, 95% confidence interval [CI]: 0.11–0.32, *P* < 0.001).

After 2 wks, there was no longer evidence of any main effects or interactions (all *P* > 0.05), with pairwise comparisons (Video v. control: *P* = 0.10, *d* = 0.11; Questions v. control: *P* = 0.87, *d* = 0.01; both interventions v. control: *P* = 0.33, *d* = 0.06; see Table 3).

### Secondary Outcomes

#### Intention to follow the treatment plan recommended by the doctor without further questioning

Immediately post-intervention, intention to follow the treatment plan for low-back pain without further questions was significantly lower for those who were presented the Video compared with those who were not, *F*(1, 1,430) = 8.92, *P* = 0.003. Pairwise comparisons showed that compared with control, there was a significant reduction in intention to follow the treatment plan without further questions for the Video (*P* = 0.02, *d* = 0.14) and both interventions (*P* = 0.04, *d* = 0.12) but not for the Questions (*P* = 0.99, *d* = 0.001). At follow-up, weak evidence of a main effect of the Video was retained, *F*(1, 1,129) = 3.08, *P* = 0.08, driven by a significant reduction in intentions when both interventions were presented compared with control (*P* = 0.016, *d* = 0.20). There was no main effect of the Questions and no interaction between the Video and the Questions at either time point.

#### Attitudes toward and preparedness for SDM

Immediately post-intervention, participants who received the Video had a more positive attitude toward SDM regarding treatment for low-back pain, χ^2^(1) = 5.38, *P* = 0.02. Pairwise comparisons showed statistical evidence of a difference in attitudes when the Video was presented (Video v. control: *P* = 0.007; both interventions v. control: *P* = 0.013) but not when the Questions were presented independently (*P* = 0.09).

The level of preparedness for SDM immediately postintervention was moderately high across all 3 groups, with no main effect of intervention group, *F*(2, 1,046) = 1.14, *P* = 0.32, and no significant pairwise differences between the groups.

#### Knowledge of patients’ healthcare rights

Most participants (83.5%) had accurate knowledge (i.e., correct response to all 4 items) of a patient’s healthcare rights at baseline (prior to exposure to interventions). After controlling for baseline knowledge, there was no evidence of a main effect of the Video, χ^2^(1) = 0.55, *P* = 0.46; Questions, χ^2^(1) = 0.23, *P* = 0.63; or their interaction, χ^2^(1) = 0.32, *P* = 0.57. Pairwise comparisons between control and active intervention arms showed no statistical evidence of a difference (all *P* > 0.05).

#### Acceptability and proactive intervention use

Acceptability of interventions and proactive intervention use (i.e., clicking on a hyperlink on the study page to access the intervention) were assessed in study arms 1 to 3 only. Acceptability was high across all arms, with more than 80% of participants in all study arms indicating that they would recommend the intervention (Video, Questions, or both) to others. However, only a small number of participants from the study in each arm proactively accessed the intervention after the study (range: 1.7%–20.8%). This was highest for the Questions arm both immediately postintervention and at 2-wk follow-up. See Appendix D.

#### Healthcare questions

We asked participants to identify what questions they would ask the clinician in the presented hypothetical clinical scenario about treatment for low-back pain. Appendix E shows the number and percentage of participants who identified healthcare questions that mapped to the Choosing Wisely 5 questions immediately and 2 wks post-intervention. Both immediately and 2 wks post-intervention, we observed a main effect of the Video (immediate: χ^2^[1] = 29.76, *P* < 0.001; 2 wk: χ^2^[1] = 11.55, *P* < 0.001) and Questions (immediate: χ^2^[1] = 244.73, *P* < 0.001; 2 wk: χ^2^[1] = 29.23, *P* < 0.001) on the total number of Choosing Wisely questions listed by participants. Compared with control, participants who were randomized to receive either intervention independently (Video: immediate: *P* < 0.001, 2 wk: *P* = 0.011; Questions: immediate: *P* < 0.001, 2 wk: *P* < 0.001) or both interventions (immediate: *P* < 0.001, 2 wk: *P* < 0.001) asked more questions that mapped to the Choosing Wisely 5 questions at both time points. There was evidence of an interaction between interventions immediately postintervention (*P* = 0.01; i.e., a greater than additive effect of combining both the Video and Questions on the number of questions that mapped to the Choosing Wisely questions). This interaction was not significant after 2 wks (*P* = 0.97).

## Discussion

This study sought to assess the impact of the consumer Choosing Wisely questions, both alone and in combination with an additional video intervention, using a randomized design. Both the Choosing Wisely Questions and SDM Preparation Video performed significantly better than control for improving participants’ intention to engage in SDM immediately post-intervention but not at 2-wk follow-up. Both interventions, whether presented independently or combined, also resulted in participants identifying significantly more questions that align with Choosing Wisely, including after 2 wks. We found some evidence that the video improved outcomes compared with control in ways that the Choosing Wisely questions alone did not, including lower intention to follow a low-value treatment plan without further questions and more positive attitude toward SDM. We also found that combining the video and Choosing Wisely questions led to a greater increase in intention to engage in SDM at immediate follow-up than presenting the Choosing Wisely questions alone. However, neither intervention changed participants’ self-efficacy to ask questions and be involved in decision-making, which was already high in this study, nor affected perceptions of preparedness to engage in SDM or knowledge of rights to be involved in healthcare decision-making.

Our findings align with and extend previous research. Building on primarily descriptive findings related to the reach of the consumer Choosing Wisely questions, this is the first study to show an impact of these questions on consumer outcomes in the context of low-value care, including on recall and intentions to engage in SDM. We also observed some modest benefits of an SDM preparation video compared with control and the Choosing Wisely questions. This aligns with suggestions that people may need additional support to be involved in decision-making (e.g., reassurance that participation would not result in retribution^12^) over and above providing tools such as question prompt lists.^25^ This is the first study to quantitatively show this in relation to the Choosing Wisely questions. Joseph-Williams et al.^12^ initially recommended that preparatory support for SDM be provided by patients’ clinicians prior to a consultation (e.g., embedded within an appointment letter). However, the video used in this study included research team members and a general practitioner who was not known to participants. While we may have observed larger impacts if clinicians provided this reassurance themselves, high-quality evidence is needed to confirm this.^26^ A generic video, such as that used in our study, also represents a more time and cost-efficient intervention model that is potentially scalable.

Despite some positive impacts, interventions in this study did not uniformly improve all outcomes, including several outcomes for which there is a theoretical relationship. Models of behavior change, for example, often assume that self-efficacy expectations will significantly increase the prediction of behavioral intentions.^27^ However, we did not observe differences in self-efficacy to ask questions or be involved in health decisions despite changes in intentions to engage in SDM. Given that it is possible that self-efficacy, which was high across all groups at baseline, was overestimated in this hypothetical study, future research is needed to explore this relationship in real-world low-value care settings. It is also possible that other unmeasured variables known to impact health behavior, such as subjective norms, may have affected intentions. This too points to an important direction for low-value care research.

There were also several variables included in this study with some level of conceptual overlap (e.g., preparedness for SDM and self-efficacy). Although outside the scope of our preplanned analysis, it would be useful to explore the predictive value of individual variables on patient outcomes in both hypothetical and clinical research settings.

Despite some significant findings, effect sizes for all interventions were small, as has been the case for other SDM interventions in low-value care contexts.^26,28^ A recent systematic review of overuse concepts found that they are difficult to grasp, sometimes received skeptically, have limited effect on behavioral intentions, and fit uncomfortably with a range of entrenched broader beliefs.^29^ This review helps to contextualize the small differences in outcomes observed in our study (including improvements in intentions to engage SDM and awareness and recall of SDM questions). There may still be value in pursuing simple, low-cost strategies such as SDM videos and prompt lists, which are immediately implementable given these possible (albeit small) benefits. Future research should continue to explore the application and impact of individual-level interventions in real-world clinical contexts alongside the implementation of macro-level strategies enacted by the government or national institutes to make low-value care inaccessible or unprofitable.^30^

### Strengths and Limitations

Interventions in this study were delivered online to a community sample using hypothetical scenarios. We considered demonstrating the evidence of impact on cognitive and affective outcomes an important first step before moving to more intensive, costly, and burdensome effectiveness evaluations in clinical settings. This study design also allowed for targeted recruitment of people with lower health literacy and enabled us to randomly allocate a large sample of participants to achieve adequate statistical power to detect small differences in outcomes. Another strength was the blinded preplanned analysis.

However, the generalizability of our findings may be limited due to the use of a hypothetical scenario, measuring intention rather than behavior, and the controlled conditions that meant that interventions were delivered in a way that diverges from how they would be delivered in the real world. Results may have also been influenced by the repeated measures, which could have led to a satisficing bias among participants, thus reducing group differences. Only 36% of participants reported a history of back pain, and so we may expect different results for clinical populations. Our study was further limited by recruiting through a social research panel. Although Dynata samples from a participant panel that is closely aligned with sociodemographic characteristics of the national population, it is possible that this sample would have had greater motivation to ask questions and be involved in health care decisions than people in the general community. Administering the interventions to assess their effectiveness in a more real-world setting may produce more accurate estimates of effect sizes.

## Conclusion

Reducing low-value health services is a complex issue, with significant clinical and health policy implications. This is the first randomized study to demonstrate that the consumer Choosing Wisely questions and a video intervention to promote question asking and engagement in SDM may improve participants’ intention to engage in SDM and support them in identifying questions that align with the Choosing Wisely campaign (with some additional benefits of the video intervention in reducing willingness to accept treatment without asking questions). Although effect sizes are small, and we did not find significant improvements on all outcomes and at all time points, the simple, low-cost nature of the interventions may make them appropriate for implementation within a suite of approaches to address low-value care at a population level.^31^ Future studies in clinical contexts will help to confirm this.

## Supplemental Material

sj-doc-2-mdm-10.1177_0272989X231184461 – Supplemental material for Randomized Trial of the Choosing Wisely Consumer Questions and a Shared Decision-Making Video Intervention on Decision-Making Outcomes Click here for additional data file. Supplemental material, sj-doc-2-mdm-10.1177_0272989X231184461 for Randomized Trial of the Choosing Wisely Consumer Questions and a Shared Decision-Making Video Intervention on Decision-Making Outcomes by Danielle Marie Muscat, Rachel Thompson, Erin Cvejic, Jenna Smith, Edward Hoi-fan Chang, Marguerite Tracy, Joshua Zadro, Robyn Lindner and Kirsten J. McCaffery in Medical Decision Making

sj-docx-1-mdm-10.1177_0272989X231184461 – Supplemental material for Randomized Trial of the Choosing Wisely Consumer Questions and a Shared Decision-Making Video Intervention on Decision-Making Outcomes Click here for additional data file. Supplemental material, sj-docx-1-mdm-10.1177_0272989X231184461 for Randomized Trial of the Choosing Wisely Consumer Questions and a Shared Decision-Making Video Intervention on Decision-Making Outcomes by Danielle Marie Muscat, Rachel Thompson, Erin Cvejic, Jenna Smith, Edward Hoi-fan Chang, Marguerite Tracy, Joshua Zadro, Robyn Lindner and Kirsten J. McCaffery in Medical Decision Making
