# Supplementary material for: Randomized Trial of the Choosing Wisely Consumer Questions and a Shared Decision-Making Video Intervention on Decision-Making Outcomes
Source: Med Decis Making. 2023 Jul 5;43(6):642–55. doi: 10.1177/0272989X231184461 (PMC10422858; doi:10.1177/0272989X231184461)

# Appendices

**Appendix A: Choosing Wisely Questions**


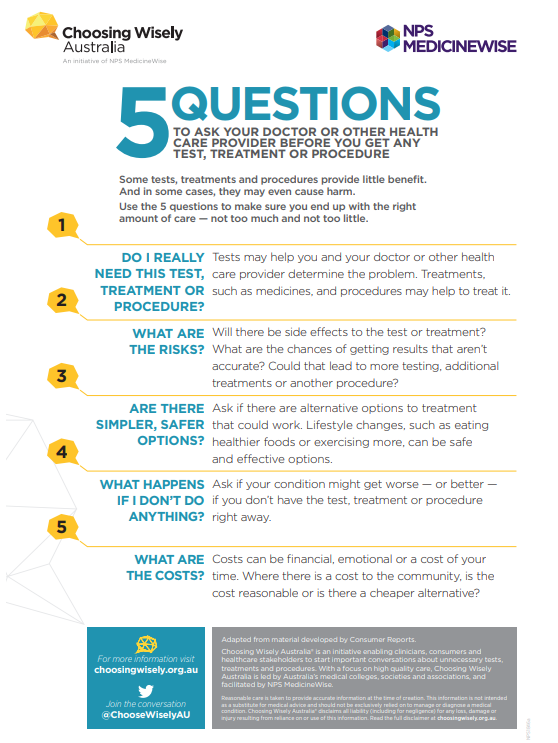


**Appendix B: Multiple Imputation of Missing Data at two-week follow-up**

Multiple imputation using chained equations (MICE) was used to impute the 301 missing values (20.9%) for the three continuous outcome measures at two-week follow-up (intention to engage in shared decision making, self-efficacy to ask questions and be involved in healthcare, and intention to follow treatment plan without further questioning). The imputation models were implemented in Stata IC v16.1 (Stata Corp, College Station, Texas, US) and comprised 30 imputed datasets. Values of the outcomes at baseline and immediate follow-up, allocated intervention and health literacy adequacy were included as auxiliary variables. The regression coefficients, standard errors and 95% confidence intervals from complete case and MICE models are displayed in the tables below.

***Supplementary Table 2.1: Self-efficacy to ask questions and be involved in healthcare***

|  | Complete Case Analysis | | | Multiple Imputation using Chained Equations (MICE) | | |
| --- | --- | --- | --- | --- | --- | --- |
| Parameter | B | SE | 95% CI | B | SE | 95% CI |
| Intercept | 32.80 | 2.098 | 28.69, 36.92 | 33.36 | 2.274 | 28.87, 37.84 |
| Intervention 1 | 2.732 | 1.548 | -0.31, 5.77 | 2.231 | 1.519 | -0.75, 5.22 |
| Intervention 2 | 1.511 | 1.586 | -1.60, 4.62 | 0.993 | 1.610 | -2.17, 4.16 |
| Intervention 1 x Intervention 2 | -3.405 | 2.232 | -7.78, 0.97 | -2.895 | 2.176 | -7.17, 1.38 |
| Health Literacy adequacy | -0.254 | 1.451 | -0.310, 2.59 | -0.465 | 1.410 | -3.23, 2.30 |
| HL x Intervention 1 | -1.488 | 2.106 | -5.62, 2.64 | -0.777 | 2.032 | -4.77, 3.21 |
| HL x Intervention 2 | -1.559 | 2.113 | -5.71, 2.59 | -1.228 | 2.093 | -5.34, 2.88 |
| HL x Intervention 1 x Intervention 2 | 2.235 | 3.023 | -3.70, 8.17 | 2.198 | 2.910 | -3.52, 7.91 |
| Self-efficacy at baseline | 0.599 | 0.023 | 0.55, 0.64 | 0.595 | 0.024 | 0.55, 0.64 |

***Supplementary Table 2.2: Intention to engage in shared decision making***

|  | Complete Case Analysis | | | Multiple Imputation using Chained Equations (MICE) | | |
| --- | --- | --- | --- | --- | --- | --- |
| Parameter | B | SE | 95% CI | B | SE | 95% CI |
| Intercept | 2.711 | 0.134 | 2.45, 2.97 | 2.710 | 0.138 | 2.44, 2.98 |
| Intervention 1 | 0.100 | 0.116 | -0.13, 0.33 | 0.081 | 0.114 | -0.14, 0.31 |
| Intervention 2 | 0.035 | 0.118 | -0.20, 0.27 | 0.032 | 0.111 | -0.19, 0.25 |
| Intervention 1 x Intervention 2 | -0.137 | 0.167 | -0.46, 0.19 | -0.091 | 0.163 | -0.41, 0.23 |
| Health Literacy adequacy | -0.050 | 0.108 | -0.26, 0.16 | -0.043 | 0.104 | -0.25, 0.16 |
| HL x Intervention 1 | 0.051 | 0.157 | -0.26, 0.36 | 0.066 | 0.153 | -0.23, 0.37 |
| HL x Intervention 2 | -0.040 | 0.158 | -0.35, 0.27 | -0.041 | 0.147 | -0.33, 0.25 |
| HL x Intervention 1 x Intervention 2 | 0.134 | 0.226 | -0.31, 0.58 | 0.093 | 0.217 | -0.33, 0.52 |
| SDM intention at baseline | 0.459 | 0.024 | 0.41, 0.51 | 0.458 | 0.025 | 0.41, 0.51 |

***Supplementary Table 2.3: Intention to follow treatment plan without further questioning***

|  | Complete Case Analysis | | | Multiple Imputation using Chained Equations (MICE) | | |
| --- | --- | --- | --- | --- | --- | --- |
| Parameter | B | SE | 95% CI | B | SE | 95% CI |
| Intercept | 4.034 | 0.284 | 3.48, 4.59 | 4.143 | 0.280 | 3.59, 4.69 |
| Intervention 1 | 0.246 | 0.271 | -0.29, 0.78 | 0.159 | 0.265 | -0.36, 0.68 |
| Intervention 2 | 0.275 | 0.278 | -0.27, 0.82 | 0.162 | 0.270 | -0.37, 0.69 |
| Intervention 1 x Intervention 2 | -0.180 | 0.391 | -0.95, 0.59 | -0.097 | 0.392 | -0.87, 0.67 |
| Health Literacy adequacy | 0.055 | 0.253 | -0.44, 0.55 | -0.108 | 0.264 | -0.59, 0.38 |
| HL x Intervention 1 | -0.922 | 0.369 | -1.65, -0.20 | -0.713 | 0.358 | -1.42, -0.01 |
| HL x Intervention 2 | -0.810 | 0.370 | -1.54, -0.08 | -0.633 | 0.359 | -1.34, 0.07 |
| HL x Intervention 1 x Intervention 2 | 0.288 | 0.530 | -0.75, 1.33 | 0.151 | 0.511 | -0.85, 1.15 |
| Intention to follow plan at baseline | 0.443 | 0.029 | 0.39, 0.50 | 0.440 | 0.030 | 0.38, 0.50 |

**Appendix C: Summative Content Analysis Additional Information**

Prior to undertaking coding, it was determined that the participant’s response did not have to replicate the question verbatim for the participant to be given a code of ‘1’. RT and JS undertook coding for the first five participants collaboratively to assist with uniformity in approach and then undertook coding for the remaining participants independently. Coders were blinded to condition allocation but not time of assessment (i.e. immediately post-intervention; 2-weeks post-intervention) when undertaking the coding. Inter-coder agreement was calculated for each of the five questions using Cohen’s Kappa and was found to be *substantial* to *almost perfect* ^32^: 0.79 for Choosing Wisely question 1, 0.89 for question 2, 0.85 for question 3, 0.92 for question 4, and 0.88 for question 5. Discrepant codes were resolved by discussion between RT, JS, and KM.

**Appendix D: Acceptability of Interventions and Proactive Intervention Use**

**Supplementary Table 3.1*.*** Acceptability of Interventions and Proactive Intervention Use, Stratified by Study Arm (1-3 only).

|  | **Study arm** | | | | |
| --- | --- | --- | --- | --- | --- |
|  | **Video (N=356)** | **Questions (N=349)** | **Both interventions (N=351)** | | |
|  |  |  |  | | |
|  | n (%) | n (%) | *Video*  n (%) | *Questions*  n (%) | *Both*  n (%) |
| Would recommend to others | 285 (80.1%) | 304 (87.1%) | 284 (80.9%) | 291 (82.9%) | 290 (82.6%) |
| Would use again | 177 (49.7%) | 304 (87.1%) | 206 (58.7%) | 271 (77.2%) | 240 (68.4%) |
| Proactively accessed the intervention – |  |  |  |  |  |
| *Immediately post-intervention* | 12 (3.4%) | 30 (8.6%) | 6 (1.7%) | 9 (2.6%) | 6 (1.7%) |
| *2-week follow-up^†^* | 28 (10.1%) | 48 (20.8%) | 24 (8.7%) | 33 (12%) | 9 (3.3%) |

*^†^ 2-week follow-up N: Preparation video (N=276), Choosing Wisely questions (N=231), Both interventions (N=275)*

**Appendix E: Healthcare Questions**

| **Supplementary Table 5.1*.*** Number (%) of responses that mapped to the Choosing Wisely 5 questions immediately, and 2-weeks post-intervention. | | | | | |
| --- | --- | --- | --- | --- | --- |
|  |  | **Video**  **(n=356)** | **Questions**  **(n=349)** | **Both interventions**  **(n=351)** | **Control**  **(n=383)** |
| **Immediately post-intervention; n (%)** | |  |  |  |  |
|  | No questions | 190 (53.4%) | 134 (38.4%) | 116 (33.0%) | 260 (67.9%) |
|  | One Question | 98 (27.5%) | 78 (22.3%) | 60 (17.1%) | 99 (25.8%) |
|  | More than one question | 68 (19.1%) | 137 (39.3%) | 175 (49.9%) | 24 (6.3%) |
| **Two-week follow-up; n (%)** | | **(n=276)** | **(n=279)** | **(n=275)** | **(n=308)** |
|  | No questions | 149 (54.0%) | 136 (48.8%) | 133 (48.4%) | 192 (62.3%) |
|  | One Question | 81 (29.3%) | 79 (28.3%) | 52 (18.9%) | 87 (28.3%) |
|  | More than one question | 46 (16.7%) | 64 (22.9%) | 90 (32.7%) | 29 (9.4%) |

**Appendix F: Choosing Wisely Scenario**


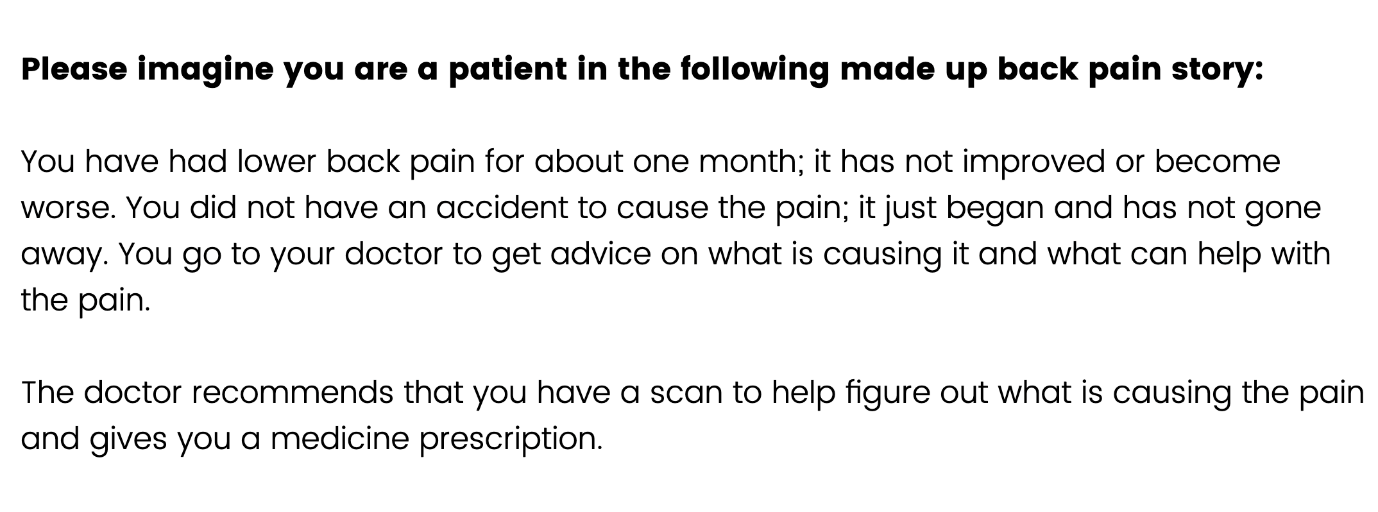

Supplement: sj-docx-1-mdm-10.1177_0272989X231184461 – Supplemental material for Randomized Trial of the Choosing Wisely Consumer Questions and a Shared Decision-Making Video Intervention on Decision-Making Outcomes [file sj-docx-1-mdm-10.1177_0272989X231184461.docx]
